# Gold coast criteria but not neurofilaments improve diagnostic sensitivity in a specialized ALS university center

**DOI:** 10.1016/j.cnp.2026.06.004

**Published:** 2026-06-12

**Authors:** Caroline Klyk, Camilla Wohnrade, Susanne Petri, Aiden Haghikia, Sonja Körner, Lars Hendrik Volk

**Affiliations:** Department of Neurology, Hannover Medical School, 30625 Hannover, Germany.

**Keywords:** Amyotrophic lateral sclerosis, Gold coast criteria, Sensitivity, Neurofilaments, Biomarkers

## Abstract

**Objective:**

Amyotrophic lateral sclerosis (ALS) is a fatal neurodegenerative disease marked by upper and lower motor neuron loss. Diagnosing ALS may still be challenging due to the absence of specific biomarkers and requires thorough clinical evaluation, comprehensive electromyography (EMG), and exclusion of differential diagnoses by laboratory analyses and imaging. The Gold Coast criteria (GCC) replaced the revised El Escorial (rEEC) and Awaji criteria (AC), simplifying ALS diagnosis and standardizing communication with patients.

**Methods:**

This retrospective study compared the sensitivity of the GCC with the rEEC and AC in a specialized neuromuscular center.

**Results:**

431 patients with suspected ALS were included, and 426 patients ultimately received an ALS diagnosis. The GCC showed higher sensitivity than both the rEEC and AC. The explorative inclusion of neurofilament levels into an extended diagnostic framework did not increase the sensitivity of rEEC and AC.

**Conclusions:**

Continued clinical use of the GCC should be considered the standard for ALS diagnosis. Careful clinical and electrophysiological examination is particularly essential in this context, supplemented by biomarkers such as neurofilaments.

**Significance:**

This retrospective real-world study demonstrates a high sensitivity of the GCC in a specialized neuromuscular clinic.

## Highlights

• Gold Coast Criteria for amyotrophic lateral sclerosis show a high sensitivity.
• Gold Coast Criteria can be safely applied in a specialized neuromuscular setting.
• Neurofilaments have no impact on the sensitivity of previous diagnostic criteria.

## 1 Introduction

Amyotrophic Lateral Sclerosis (ALS) is the most common motor neuron disease in adults, with an incidence of 1–2 cases per 100,000 people annually (Chiò et al., 2013). Despite this low incidence, ALS imposes a significant social and economic burden due to its progressive, care-intensive, and fatal nature, in most cases compounded by the lack of curative treatments. Timely and accurate diagnosis is critical, as delays prolong the diagnostic process and the uncertainty for the affected individuals, and in some cases the initiation of disease modifying gene therapy.

ALS incidence increases with age, peaking between the ages 60 and 69 with an average age of onset in the mid-to-late 50s. (Feldman et al., 2022) The estimated prevalence is 6 cases per 100.000 and the average survival is 3–4 years post-diagnosis (Talbott et al., 2016). The disease spans a spectrum of subtypes, distinguished by region of onset, such as bulbar, limb, or thoracic, progression patterns and involvement of upper and lower motor neurons (Vidovic et al., 2023). While the underlying neuropathophysiology—comprising motor neuron degeneration, protein aggregation, and inflammation, —remains incompletely understood, it unifies ALS as a disease spectrum (Ravits and La Spada, 2009).

Currently, no curative treatment exists for sporadic ALS. The first disease-modifying drug, riluzole, a sodium channel blocker, has been available in Germany since 1996. A combined meta-analysis of the first two randomized clinical trials demonstrated a benefit of survival of 2 to 3 months, though real-world data suggest an extended survival period between 6 and 19 months across all ALS subtypes (Corcia et al., 2024). Beyond pharmacological treatment, supportive therapies such as physical and speech therapy play a crucial role in maintaining autonomy and improving quality of life (Peseschkian et al., 2021).

**Fig. 1:**
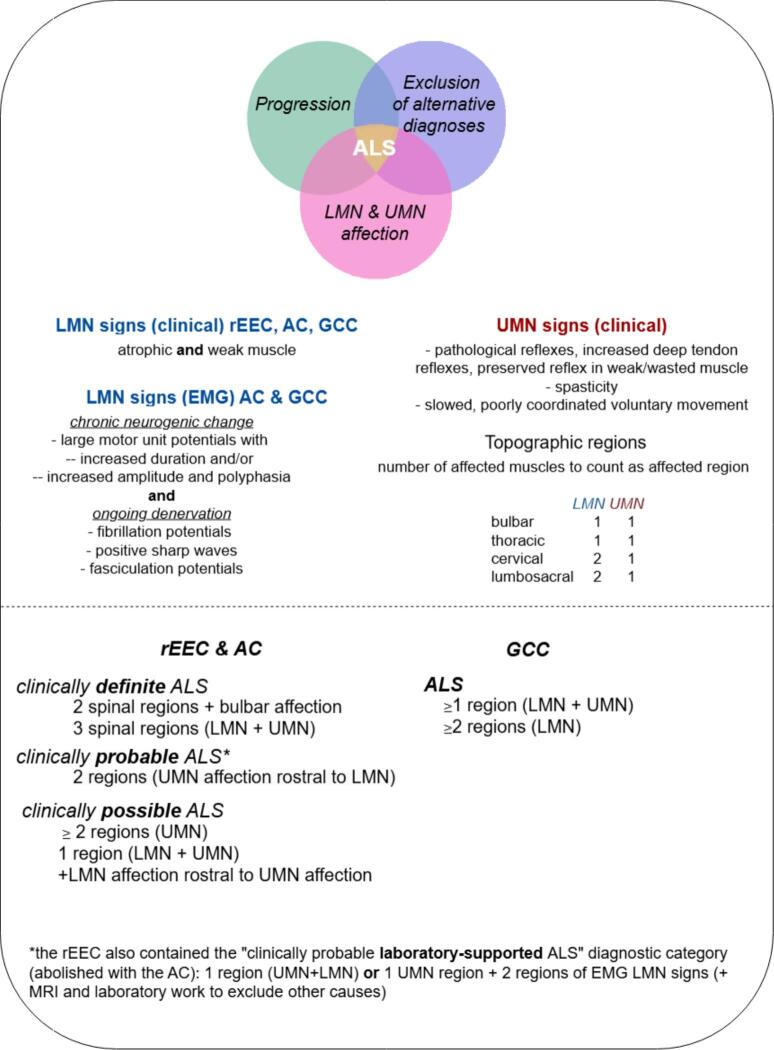
Overview of the diagnostic criteria for Amyotrophic lateral sclerosis. AC: Awaji criteria. ALS: Amyotrophic lateral sclerosis. EMG: electromyography. GCC: Gold Coast criteria. LMN: lower motor neuron. rEEC: revised El Escorial criteria.UMN: upper motor neuron. (For interpretation of the references to colour in this figure legend, the reader is referred to the web version of this article.)

Diagnostic delays in ALS, averaging 12–18 months, often result from the heterogenous clinical presentations and the limitations of existing diagnostic criteria (Paganoni et al., 2014). Several diagnostic criteria have been developed to improve ALS diagnosis, including the revised El Escorial criteria (rEEC), the Awaji criteria (AC), and the Gold Coast criteria (GCC), as detailed in Fig. 1. Each of these criteria requires three fundamental conditions: (1) evidence of disease progression, (2) the completion of differential diagnostics, and (3) the presence of upper and/or lower motor neuron involvement.

The El Escorial Criteria (EEC) (Brooks, 1994) and their revised version, the rEEC (Brooks et al., 2000), were primarily designed for research but were widely used in clinical practice due to a lack of alternatives. The rEEC classified patients into *definite ALS*, *probable ALS*, *clinically probable laboratory-supported ALS*, and *possible ALS*, eliminating the EEC's previous *suspected ALS* category.

However, these classifications presented challenges: patients categorized as *possible ALS* often did not transition between categories before the disease reached its terminal phase, illustrating the limited prognostic differentiation (Traynor et al., 2000). Furthermore, the different subcategories suggested a sequential disease progression, which was misleading for both patients and clinicians. Communicating a diagnosis of *possible ALS* was particularly challenging, as the diagnostic probability strongly suggested ALS, yet the categorization reflected contradictory results, leading to repeated diagnostic evaluations. Additionally, the complexity of the EEC and rEEC led to poor inter-rater variability (Johnsen et al., 2019). Moreover, the rEEC's structure limited early-stage patients' participation in clinical trials, thereby impacting drug efficacy assessments (Carvalho and Swash, 2009; Okita et al., 2011).

To address these issues, the AC were introduced in 2008, recognizing electromyography (EMG) findings as equivalent to clinical observations (de Carvalho et al., 2008). This modification improved diagnostic sensitivity and allowed earlier diagnosis, enabling recruitment of early disease stage patients into clinical trials (Costa et al., 2012). However, despite these advantages, the AC remained complex and posed communication challenges, even after eliminating the *clinically probable laboratory-supported ALS* category.

In response to these limitations, the GCC were introduced in 2019 (Shefner et al., 2020) to streamline ALS diagnosis. The GCC eliminated subcategories, classifying patients simply as having *ALS* or *no ALS*. Diagnosis requires evidence of progressive motor neuron dysfunction, with signs of upper motor neuron (UMN) and lower motor neuron (LMN) involvement in a single anatomical region or LMN signs in at least two regions. Following the introduction of the GCC, several studies have evaluated their sensitivity and specificity in comparison to the rEEC and AC. Retrospective analyses from European and Chinese ALS cohorts demonstrated higher diagnostic sensitivity of the GCC (Pugdahl et al., 2021, Shen et al., 2021). Importantly, the increased sensitivity did not compromise specificity (Turner and Group, 2022).

Beyond clinical signs and EMG, no additional diagnostic biomarkers have been included into any of the diagnostic criteria. However, supplementary diagnostic methods are frequently used to support ALS diagnosis. For instance, ultrasound can visualize fasciculations, providing evidence of LMN involvement (Tsugawa et al., 2018). Neurofilament light chain (NfL) and phosphorylated neurofilament heavy chain (pNfH) in serum and cerebrospinal fluid increase and remain significantly elevated in ALS patients, reflecting neuroaxonal damage, and have been confirmed as valuable diagnostic and prognostic biomarkers (Meyer et al., 2024; Meyer et al., 2023; Steinacker et al., 2016). Retrospective studies also highlighted their utility in distinguishing ALS from ALS mimics such as multifocal motor neuropathy (MMN) (Wohnrade et al., 2024).

This monocentric, retrospective study pursues a sensitivity-focused approach in evaluating the GCC in a high-pretest-probability cohort aiming to support its use for earlier diagnosis and timely therapeutic intervention. Given their potential to enhance diagnostic accuracy, we explored the integration of neurofilaments into an expanded diagnostic framework.

## 2 Materials and methods

### 2.1 Patient cohort and data collection

To enhance the completeness and transparency of reporting in diagnostic accuracy studies, the STARD 2015 guideline (Bossuyt et al., 2015) was adhered to during the conduction of this sensitivity-focused study. In our retrospective monocenter study, we consecutively included patients aged 18 years or older with suspected ALS, who were referred to the Department of Neurology at Hannover Medical School (Hannover, Germany) for further diagnostic testing. Additional inclusion criteria were availability of comprehensive EMG data and further diagnostic tests to rule out other underlying neurological diseases in accordance with diagnostic guidelines at the respective time, such as laboratory analyses, nerve conduction studies, brain and spinal magnetic resonance imaging (MRI) either during the in house stay or beforehand. Patients were either referred to our clinic by their respective (local) neurologists, by our emergency room, or by our outpatient clinic specialized in neuromuscular diseases. Patient data was collected between August 2010 and July 2023. If available, follow-up reports were used for additional confirmation in each group. As not all patients continued their treatment at Hannover Medical School, follow-up data could not be obtained for all individuals. All tests were performed during the initial inpatient admission, and no disease-modifying treatments were initiated before diagnosis. Informed consent was waived by the local ethics committee. Anonymization and data processing were conducted in accordance with the local ethics committee (No. 11397_BO_K_2024).

For our analysis, we collected the study data from the hospital information system SAP IS-H (SAP SE, Walldorf, Germany) and reviewed the medical reports, written results of EMG studies and laboratory testing. We included the following parameters:

1) socio-demographic data (age, gender)
2) disease-specific data (age at onset, region of clinical onset, duration from onset until diagnosis, result of genetic testing for ALS-associated mutations, subtype if applicable)
3) clinical examination findings categorized in the presence or absence of UMN and LMN signs in the bulbar, cervical, thoracic, and lumbar region (i.e., presence of upper UMN signs such as slow tongue movement, hyperreflexia or brisk deep tendon reflexes in atrophic muscles, positive pyramidal tract signs, spasticity; presence of LMN signs such as atrophic paresis, tongue atrophy, and fasciculations)
4) EMG findings categorized in presence or absence of ongoing denervation and/or chronic neurogenic change (i. e. ongoing denervation defined as fibrillation potentials, positive sharp waves, and fasciculation potentials; chronic neurogenic change defined as large motor unit potentials, increased duration, and thinned-out interference pattern). Throughout the inclusion period a standardized EMG protocol was implemented at Hannover Medical School, covering at least one proximal and one distal limb muscle on one side as well as one proximal or distal muscle on the other side (thus, covering all four limbs), the paravertebral muscles, and the tongue (translating to the lumbar, thoracal, cervical and bulbar region). Per muscle two insertion sites were evaluated and, for the assessment of chronic neurogenic change, at least ten motor unit potentials during slight contraction were recorded. In each patient all four EMG regions were examined with rare exceptions. Sporadically patients refused EMG of the tongue (bulbar region) or could not relax sufficiently during EMG of the paravertebral muscles (thoracal region) resulting in missing data. In the cervical and lumbar regions, ongoing denervation and chronic neurogenic change were defined as abnormalities, while in the bulbar and thoracal regions only the presence of ongoing denervation was accounted for.
5) Levels of neurofilaments in serum and cerebrospinal fluid, if applicable. Serum NfL was measured using the Ella automated immunoassay platform, and cerebrospinal fluid (CSF) pNfH was measured using enzyme-linked immunosorbent assay (ELISA).
6) Fulfillment of the diagnostic criteria rEEC, AC, and GCC at the time of inpatient diagnosis was assessed based on the data collected. For this assessment, the authors were blinded to the final diagnosis (reference standard).

The OPM classification of ALS phenotypes recently proposed by Meyer et al. aims to replace current terms such as primary lateral sclerosis (PLS), progressive bulbar paralysis (PBP), progressive muscle atrophy (PMA) or Flail-Arm-syndrome (FAS), to improve study comparability and enable a more precise prognostic assessment. The OPM classification is based on the three components O (onset), the region of disease onset, P (propagation), the spatial and temporal spread of motor symptoms, and M (motor neuron dysfunction), the degree of involvement of UMN and LMN (Meyer et al., 2025). However, for the purposes of this study, the OPM classification was not applied, as subtype assignment was based on retrospective medical records and to maintain consistency with previously published studies. For this study, the PLS and PMA subtypes are considered as ALS phenotypes.

### 2.2 Sensitivity and specificity analysis

The combination of a history of progression and thorough diagnostic examinations to exclude mimicking disorders served as a reference standard. Further, individual clinical aspects such as for example the lack of response to immunomodulatory treatment or a certain progression pattern were taken into consideration. Thus, the final diagnosis was not primarily based on the fulfillment of either of the diagnostic criteria. The established diagnosis, as documented in the medical records, was reviewed by two experienced neurologists (authors CW and LHV) regarding the plausibility of the diagnosis. Indeterminate test results, such as unclear EMG patterns or inconclusive clinical findings, were reviewed. Patients were only included in the analyses if sufficient clinical and diagnostic criteria were met or clarified through follow-up data. In cases with ambiguous results and no follow-up, the cases were excluded from the final accuracy analysis. Patients with typical clinical findings for ALS, advanced diagnostic and laboratory findings consistent with ALS diagnosis, and sufficient exclusion of mimicking disorders were considered as ALS. Patients with a differential diagnosis other than ALS were considered non-ALS.

To test the diagnostic utility of the different diagnostic criteria, we then calculated the sensitivity and specificity of the rEEC, AC, and GCC. Given that previous studies included patients with suspected ALS and therefore demonstrated a considerable high pretest-probability between 69 and 98%, we decided to pursue a sensitivity-focused analysis of the diagnostic criteria (Hannaford et al., 2021, Pugdahl et al., 2021, Shen et al., 2021). Therefore, as there were only a limited number of non-ALS patients, specificity could not be assessed reliably, and specificity values were excluded from further analysis. Patients who met the diagnostic criteria of the GCC were considered as ALS. Since the GCC are dichotomous, patients who did not fulfill the GCC were considered as not diagnosed with ALS. Regarding the rEEC and AC, we used two different approaches determining ALS diagnosis for comparison with previous studies.

For the first or *traditional* approach, ALS patients were considered diagnosed with ALS by fulfilling the categories *definite ALS* and *probable ALS*, and *probable laboratory supported ALS* for rEEC. Patients classified as *possible ALS* and patients who did not fulfill the rEEC were considered as not diagnosed with ALS according to the rEEC. Similarly, patients classified as *possible ALS* using the AC and patients who did not fulfill the AC for ALS diagnosis were considered not diagnosed with ALS. For the alternative and more practical approach, ALS diagnosis was established when patients fulfilled any diagnostic category using either rEEC or AC (further termed rEEC possible and AC possible for discrimination). Only patients, who did not fulfill any diagnostic category, were considered not diagnosed with ALS.

Calculation of sensitivity was additionally performed in different subgroups regarding region of disease onset, disease duration (the patients were divided into two groups with a disease duration of more or less than 18 months to align with previously conducted studies), and ALS subtype.

**Fig. 2:**
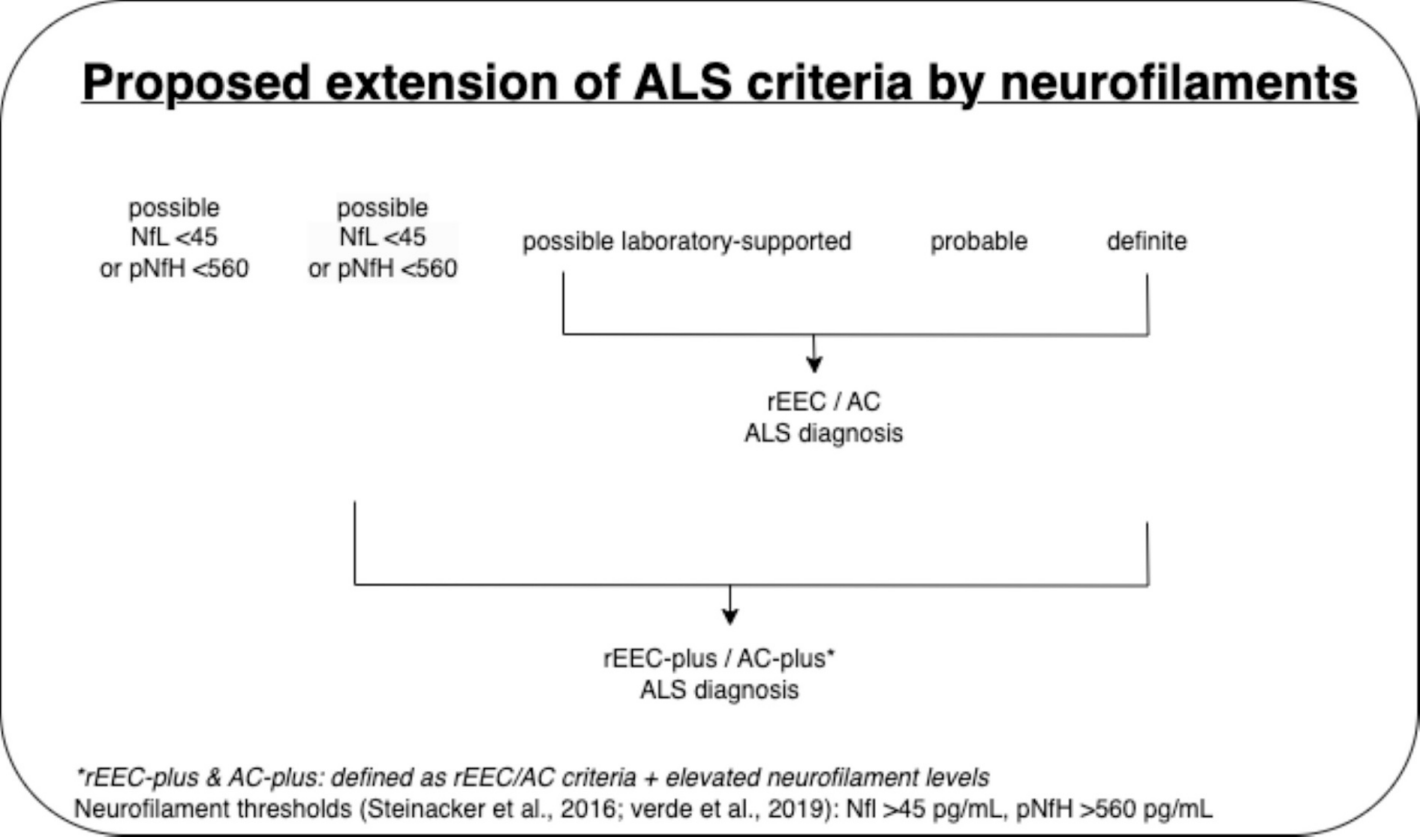
Exploratory model of a diagnostic extension for ALS diagnosis including neurofilaments. AC: Awaji criteria. ALS: Amyotrophic lateral sclerosis. GCC: Gold Coast criteria. NfL: Neurofilament light chain. pNfH: phosphorylated neurofilament heavy chain. rEEC: revised El Escorial criteria. (For interpretation of the references to colour in this figure legend, the reader is referred to the web version of this article.)

In addition to the primary assessment of diagnostic criteria, we analyzed data from patients for whom neurofilament levels were available. In an exploratory hypothesis generating approach we assessed the impact of neurofilaments as a diagnostic marker and introduced two new categories: revised El Escorial plus (rEEC-plus) and Awaji-plus (AC-plus), which represent the original diagnostic criteria supplemented by the integration of neurofilament levels, as seen in Fig. 2. For both rEEC- and AC-plus criteria, the category *possible ALS* was considered as diagnosed with ALS, along with the categories *definite*, *probable*, and *possible laboratory supported*, if neurofilament levels were elevated. As there were no non-ALS cases with available neurofilament values this analysis was restricted to sensitivity and does not allow conclusions about overall diagnostic accuracy. Neurofilament thresholds were set at >45 pg/ml for serum NfL and > 560 pg/ml for cerebrospinal fluid pNfH, based on previously established values (Steinacker et al., 2016; Verde et al., 2019). The processing of serum and CSF samples followed established protocols (Wohnrade et al., 2024). We did not create a Gold Coast (GCC)-plus category, as the particularly appealing feature of the GCC is their dichotomous structure, which we did not want to complicate. No ALS diagnosis was assigned solely based on elevated neurofilaments.

### 2.3 Statistical analysis

For statistical analysis, we used IBM SPSS Statistics Version 29 (IBM Corporation, Armonk, NY, USA). Sensitivity and specificity for all criteria subsets were calculated, and sensitivity is presented with 95% confidence intervals (CI). McNemar's test was applied to test for differences in sensitivity of the GCC compared to either rEEC or AC and rEEC possible or AC possible, respectively. Due to the exploratory character of the subgroup analysis, no correction for multiple testing was conducted. *P*-values <0.05 were considered significant. Because of the retrospective design of our study, all eligible patients were included. Post-hoc power analysis using the program G*Power (Faul et al., 2007) demonstrated a power of 99.1%. Although specificity was calculated, the limited number of non-ALS cases resulted in wide confidence intervals, which were not shown or interpreted in detail due to insufficient reliability. No imputation of missing data was performed.

## 3 Results

### 3.1 Patient cohort and clinical characteristics

**Fig. 3:**
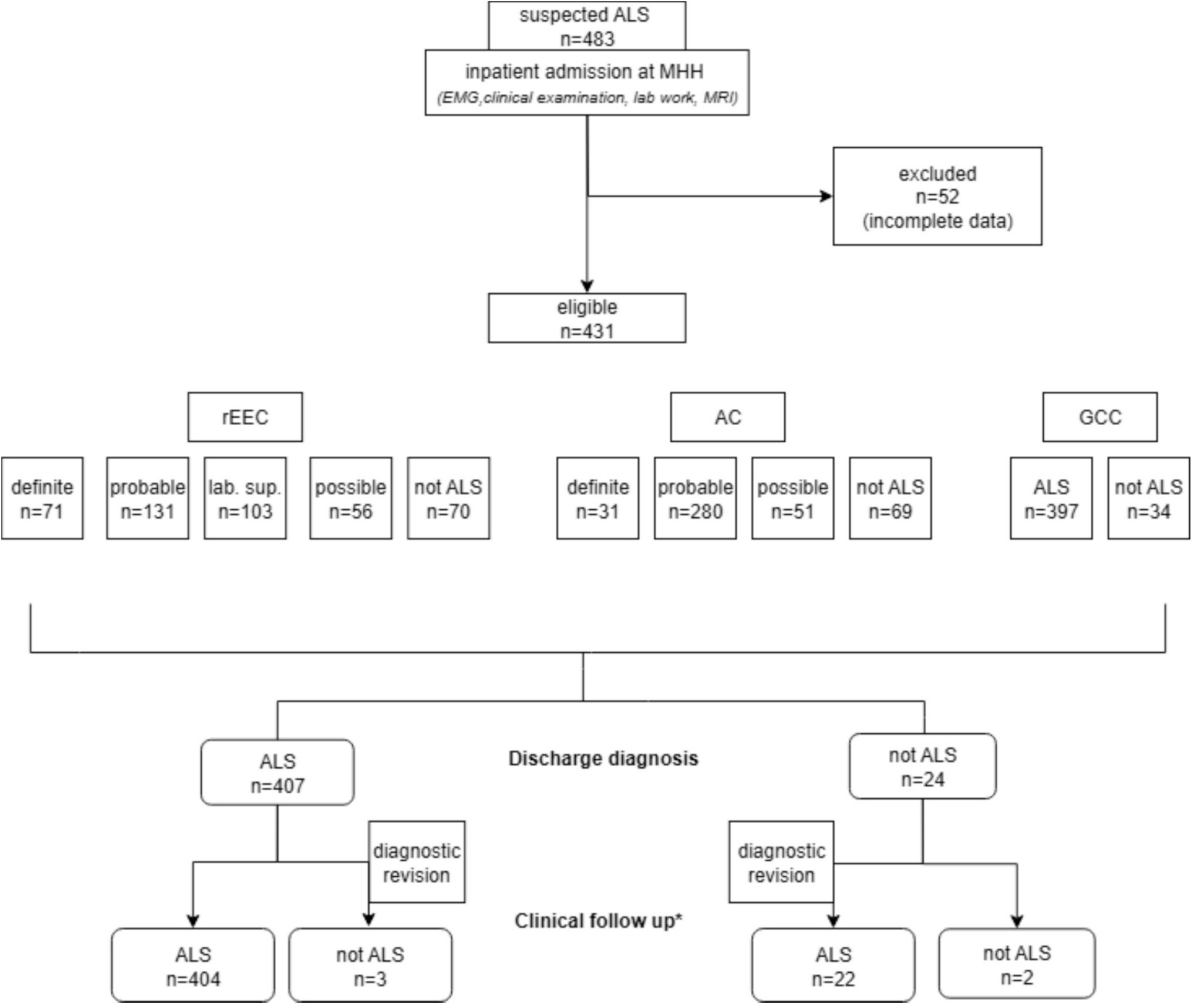
Flow diagram of patients and their diagnosis after their first inpatient stay and follow up according to the different diagnostic criteria. ALS: Amyotrophic lateral sclerosis. GCC: Gold coast criteria. MHH: Hannover Medical School. rEEC = revised El Escorial criteria. *if available. (For interpretation of the references to colour in this figure legend, the reader is referred to the web version of this article.)

In total, 483 patients were eligible for this study. After exclusion of 52 patients for not meeting the inclusion criteria (i.e., because of missing clinical examination, missing EMG data), 431 patients were included for further analysis (Fig. 3). 407 patients were diagnosed with ALS at the time of data collection, specifically during their initial inpatient admission at our clinic. Out of these, three patients underwent a revision of their diagnosis during further disease course. In contrast, 22 patients who had not been diagnosed with ALS during their initial hospitalization were later found to be diagnosed with ALS in the course of disease progression.

Of the 431 patients (238 female, 193 male), 426 patients ultimately received an ALS diagnosis. Only five patients were not diagnosed with ALS. The diagnoses of these patients were a potential hereditary spastic paraplegia (*n* = 1), histopathologically confirmed inclusion body myositis (IBM) (n = 1), immune-mediated polyneuropathy (*n* = 2), and brachial plexus neuritis (n = 1). Demographics are shown in Table 1. The average age of onset of ALS patients was 64 years (range 23 to 87 years). The median disease duration from symptom onset to diagnosis in ALS patients was 12 months (range 1–243 months). Spinal onset was most common in ALS patients (cervical onset: 29.8%, lumbar onset: 33.8%), while almost a third presented with bulbar symptoms. At the time of diagnosis, most ALS patients demonstrated a classical ALS phenotype (70.0%).

**Table 1:** Demographic and clinical data from ALS and non-ALS patients. ALS: Amyotrophic Lateral Sclerosis. ALSFRS-R: revised Amyotrophic Lateral Sclerosis Functional Rating Scale. FAS: Flail-arm syndrome. PBP: progressive bulbar paralysis. PMA: Progressive Muscular Atrophy. PLS: Primary Lateral Sclerosis.

	All patients *n* = 431	ALS *n* = 426	Non-ALS *n* = 5
Age at onset (years), median (min-max)	64 (23–87)	64 (23–87)	76 (42–78)
Female/Male (%)	238/193 (55.2/44.8)	234/192 (54.9/45.1)	1/4 (20.0/80.0)
Disease duration at diagnosis (months), median (min-max)	12 (1–243)	12 (1–243)	13 (8–240)
Onset region (%) Bulbar Cervical Thoracic Lumbal	143 (33.2) 128 (29.7) 13 (3.0) 147 (34.4)	142 (33.3) 127 (29.8) 13 (3.1) 144 (33.8)	1 (20.0) 1 (20.0) / 3 (60.0)
Subtype (%) PBP FAS PMA PLS classic other	35 (8.1) 27 (6.3) 26 (6.0) 18 (4.2) 299 (69.4) 26 (6.0)	35 (8.2) 27 (6.3) 26 (6.1) 17 (4.0) 298 (70.0) 23 (5.4)	/ / / 1 (20.0) 1 (20.0) 3 (60.0)
ALSFRS-R at diagnosis, median (min-max)	40 (10–47)	40 (10–47)	43.5 (35–45)

### 3.2 Sensitivity & subgroup analysis

Sensitivity of the diagnostic criteria (rEEC, AC, and GCC) were compared as shown in Table 2A.

**Table 2:** Diagnostic sensitivity of the diagnostic criteria sets in the total cohort (A) and in patients with available neurofilament values (B). All patients fulfilling the rEEC possible criteria demonstrated elevated serum NfL and/or CSF pNfH. McNemar's test was applied to test for differences in sensitivity compared to the GCC and are demonstrated with 95% confidence intervals. P-values <0.05 were considered significant. AC: Awaji criteria. AC poss: Awaji criteria including the possible category. AC-plus: Awaji criteria with incorporated neurofilament values. CI: confidence intervals. FP: false positive. FN: False negative. GCC: Gold Coast criteria. rEEC: revised El Escorial criteria. rEEC. poss: revised El Escorial criteria including the possible category. rEEC-plus: revised El Escorial criteria with incorporated neurofilament values. Se: Sensitivity. TN: True negative. TP: true positive.

A	Total cohort						
Diagnostic criteria	TP	FP	TN	FN	Se (%)	95%CI	p-value
GCC	394	3	2	32	92.5	89.6–94.6	
AC	309	2	3	117	72.5	68.1–76.6	<0.001
AC poss	359	3	2	67	84.3	80.5–87.4	<0.001
rEEC	303	2	3	123	71.1	66.6–75.2	<0.001
REEC poss	358	3	2	68	84.0	80.3–87.2	<0.001

**B**	**Neurofilament cohort**						
GCC	124	0	0	3	97.3	93.3–99.2	
AC	99	0	0	28	78.0	70.0–84.3	<0.001
AC poss	114	0	0	13	89.8	83.3–93.9	0.021
AC-plus	113	0	0	14	89.0	82.3–93.3	0.007
rEEC	97	0	0	30	76.4	68.3–82.9	<0.001
rEEC poss	113	0	0	14	89.0	82.3–93.3	0.013
rEEC-plus	113	0	0	14	89.0	82.3–93.3	0.013

Among the total cohort, the GCC had the highest sensitivity at 92.5% (95% CI: 89.6–94.6%), surpassing the previous diagnostic criteria AC at 72.5% (95% CI: 68.1–76.6; *p* < 0.001) and rEEC at 71.1% (95% CI: 66.6–75.2%; p < 0.001). The GCC even outperformed the rEEC and AC when the *possible ALS* category was included: rEEC possible 84.3% (95% CI: 80.5–87.4; p < 0.001), and AC possible 84.0% (95% CI: 80.3–87.2%; p < 0.001), respectively.

For patients with a disease duration shorter than 18 months, the GCC outperformed all other diagnostic criteria with a sensitivity of 93.0% (95% CI: 89.5–95.4%) (table S1). There was no difference in sensitivity for ALS patients with a disease duration longer than 18 months when comparing the GCC (91.3%, 95% CI: 85.0–95.1%) to the AC and rEEC including the *possible ALS* category (AC possible: 81.7%, 95% CI: 74.1–87.5%; *p* = 0.052, and rEEC possible: 81.7%, 95% CI: 74.1–87.5%; p = 0.052) (table S1). Considering patients with limb cervical and lumbar onset, the GCC displayed the highest sensitivity at 96.1% (95% CI: 91.1–98.3%) and 93.1% (95% CI: 87.7–96.2%), respectively (table S1). However, in patients with bulbar onset, the sensitivity of the AC possible criteria was highest (94.4%, 95% CI: 89.3–97.1%) and slightly better than the GCC (88.0%; 95% CI: 81.7–92.4%; *p* = 0.049) (table S1). In patients with predominant LMN involvement, such as FAS and PMA, the GCC outperformed the other diagnostic criteria (FAS: 96.3%, 95% CI: 81.7–99.3% and PMA: 96.2%, 95% CI: 81.1–99.3%) (table S1). In contrast, in patients with a predominant UMN involvement, the sensitivity of the GCC was significantly lower compared to both AC possible (35.3% vs. 82.4%, *p* = 0.004) and rEEC possible (35.3% vs. 76.5%, *p* = 0.021).

### 3.3 Neurofilaments

There was no data available on neurofilament levels in non-ALS patients, mainly because standard neurofilament testing was not established in our clinic at the time of diagnostic assessment of these patients.

The average neurofilament values in ALS patients varied depending on the subtype and clinical onset (Table 3). The highest serum NfL values of 330.88 pg/ml were measured in PBP, followed by PMA with 210.33 pg/ml and FAS with 199.2 pg/ml. In contrast, the PLS subtype showed the lowest NfL value of 65.0 pg/ml. The highest pNfH values in CSF were found in patients with FAS at 3119.17 pg/ml and the PBP at 2991.13 pg/ml, while the PLS group had the lowest pNfH values at 1342.75 pg/ml.

**Table 3:** Neurofilament levels. and clinical data from ALS patients. Neurofilament values are depicted as mean and standard error of means. ALS: Amyotrophic Lateral Sclerosis. ALSFRS-R: revised Amyotrophic Lateral Sclerosis Functional Rating Scale. FAS: Flail-arm syndrome. NfL: Neurofilament light chain. PBP: progressive bulbar paralysis. pNfH: phosphorylated neurofilament heavy chain. PMA: Progressive Muscular Atrophy. PLS: Primary Lateral Sclerosis. SEM: standard error of means.

		Serum NfL mean value (pg/ml) [SEM] n = 123	CSF pNfH mean value (pg/ml) [SEM] n = 115
Total		140.55 (13.45)	2795.74 (242.96)
	ALS	140.55 (13.45)	2795.74 (242.96)
	No ALS	/	/
Onset	bulbar	193.16 (30.44)	2784.07 (344.74)
	cervical	128.5 (18.8)	2705.54 (461.96)
	thoracic	62.00 (9.34)	1043.83 (302.04)
	lumbal	105.05 (14.49)	3161.97 (520.85)
Subtype	PBP	330.88 (127.79)	2991.13 (1349.2)
	FAS	199.2 (64.71)	3119.17 (1378.73)
	PMA	210.33 (119.15)	2649.00 (1550.48)
	PLS	65.00 (19.15)	1342.75 (587.01)
	classic	122.65 (9.81)	2772.43 (252.55)
	other	113.67 (53.72)	4100 (1938.87)

Serum NfL values also differed according to the region of clinical onset. The bulbar onset patients had the highest NfL values at 193.16 pg/ml, followed by the cervical onset group with 128.5 pg/ml, and the lumbar onset with the lowest values of 105 pg/ml. Regarding CSF pNfH values, the lumbar onset patients showed the highest value of 3161.97 pg/ml, followed by the bulbar onset ones with 2784.07 pg/ml and the cervical onset group with 2705.54 pg/ml.

### 3.4 Comparison between rEEC-plus, AC-plus and GCC

As shown in Table 2B, within the cohort with available data on neurofilaments (Serum NfL: *n* = 123, CSF pNfH: *n* = 115), the inclusion of neurofilament values led to an increased number of correctly diagnosed ALS cases compared to the use of rEEC 113 vs. 97 true positives) and AC (114 vs. 99 true positives). However, the sensitivity of the AC-plus (89.0%, 95% CI: 82.3–93.3%) and rEEC-plus (89.0%, 95% CI: 82.3–93.3%) was comparable to the sensitivity of AC possible (89.8%, 95% CI: 83.3–93.9%) and rEEC possible (89.0%, 95% CI: 82.3–93.3%). The highest sensitivity was still observed with the GCC, reaching 97.3% (95% CI = 93.3–99.2%).

### 3.5 False positive and false negative cases with GCC

In the total cohort, there were only three false-positive diagnosed patients when the GCC were used. The first patient was a 78-year-old male patient with a 12-month history of gait disorder due to progressive weakness and muscle wasting as well as difficulty in swallowing. A muscle biopsy ultimately revealed lymphocytic intramuscular infiltrates and rimmed vacuoles, leading to the diagnosis of IBM. The second patient was a 67-year-old male with an 18-month history of speaking difficulty. Electrodiagnostic testing and sural nerve biopsy revealed a demyelinating neuropathy. After positive treatment effects of both intravenous steroid pulse therapy and intravenous immunoglobulin treatment, the diagnosis of multifocal acquired demyelinating sensory and motor neuropathy was established. The course of the disease remained stable under treatment for more than 9 years after initial suspicion of ALS. The last patient, a 77-year-old male, suffered from muscle wasting of the shoulder girdle of the right side and weakness of his right arm for 13 months. No clinical progression was observed four years after diagnosis, and a residual brachial plexus neuropathy was diagnosed.

Among the 32 false-negative patients in the total cohort, most patients had a bulbar onset (50%) and predominant UMN signs, with more than half displaying only UMN involvement (58.8% without LMN involvement). The median age (65, range 36–82), disease duration (11.5 months, range 3–240 months) and sex ratio (1.1) were comparable to the total cohort.

Considering the ALS patients with available data on neurofilaments, there were only 3 false-negative patients after applying the GCC. The first patient, a 78-year-old female, had a 3-month history of progressive speaking disorder and difficulty in swallowing, and weight loss. No weakness was evident, but clinical examination revealed ubiquitously enhanced muscle tone and brisk reflexes. EMG demonstrated solely fasciculations. Neurofilament levels were strongly increased (serum Nfl: 727.0 pg/ml; CSF pNfH >12.000 pg/ml). The patient fulfilled the possible ALS category applying the AC and rEEC, respectively. The second patient was a 51-year-old female patient with a 2-year history of progressive difficulty in speaking and swallowing and involuntary laughing and crying. Clinical examination identified a pseudobulbar affect, dysarthria, brisk reflexes and spasticity. EMG displayed no LMN involvement. Both serum NfL (105 pg/ml) and CSF pNfH (3072 pg/ml) were elevated. According to AC and rEEC, the patient was classified as *possible ALS*. The third patient, a 42-year-old female, had a 2-year history of gait disturbance. Clinical examination revealed distal limb weakness with brisk reflexes and positive pyramidal signs. Neither clinical examination nor EMG demonstrated LMN signs. Neurofilament levels were in the normal range (serum NfL: 5 pg/ml; CSF pNfH <188.0 pg/ml). The patient was classified as *possible ALS* according to AC and rEEC.

## 4 Discussion

The present study demonstrates improved sensitivity of the GCC compared to the rEEC and AC in a specialized neuromuscular clinic setting. Even when considering neurofilaments as the here defined rEEC-plus and AC-plus, the GCC exhibit a higher sensitivity. Our data support the notion that clinical signs and EMG findings are still essential diagnostic tools in ALS diagnosis.

A total of 431 patients were included in our monocenter study. Gender distribution as well as the age of onset and time to diagnosis reflect general ALS epidemiology (Körner et al., 2011; Li et al., 2017). Similarly, the distribution of onset regions in our cohort—33.3% bulbar and 33.8% lumbar—along with the prevalence of classic ALS at 70%, aligns with findings from other study populations (Wijesekera, 2009). Among the non-ALS patients, alternative diagnoses included typical ALS mimics such as hereditary spastic paraplegia, immune-mediated polyneuropathies, and IBM, further underlining the diagnostic challenges in early-stage ALS. The rare thoracic onset was not found in the non-ALS patients in our cohort. Disease severity was assessed using the ALS Functional Rating Scale-Revised (ALSFRS-R), with a median score of 40 (range 10–47) in ALS patients, reflecting a wide spectrum of disease progression at the time of diagnosis. The ALSFRS-R score was slightly higher in non-ALS patients (median of 43.5). This difference reflects slower disease progression and less functional impairment in non-ALS individuals.

Our study shows that the sensitivity of the GCC is consistently higher than the sensitivity of the rEEC and AC in all ALS subtypes but PLS, which is in line with previous findings regarding the diagnostic criteria for ALS (Pugdahl et al., 2021, Shen et al., 2021). The increase in sensitivity was particularly pronounced in patients with limb cervical onset, limb lumbar onset, FAS, and PMA subtypes. Hannaford and colleagues reported a PMA subtype in 8.9% of ALS patients and a high sensitivity of 94% using the GCC, while most PMA patients failed to meet the AC (58%) or rEEC (61%) (Hannaford et al., 2021). Our findings are in line with these results, with a sensitivity of 96.3% for the PMA subtype using the GCC. Both the AC and rEEC failed to correctly classify most patients with a PMA subtype: Only two patients fulfilled the AC possible, while four patients were correctly diagnosed according to rEEC when the *possible ALS* category was included. Sensitivity for the PMA subtype is higher because the GCC incorporate the affection of LMN involvement in at least two regions, whereas rEEC and AC do not allow ALS diagnosis solely based on LMN involvement. The increased sensitivity of the GCC in patients with limb-onset ALS aligns with the findings of Shen et al., who observed similar results in their single-center study conducted within a Chinese population (Shen et al., 2021). Therefore, the GCC demonstrated a good applicability, especially for cases with suspected ALS with LMN predominant phenotypes. In contrast, the sensitivity for the PLS phenotype was significantly lower, as the GCC no longer permit a diagnosis based solely on UMN signs, which was still applicable under the rEEC and AC. In this regard, it should be taken into account that PLS has historically been considered an independent disease distinct from ALS with its own diagnostic criteria (Turner et al., 2020). As the GCC were designed to simplify ALS diagnosis and not intended to cover the whole motoneuron disease spectrum, the lower sensitivity of the GCC in PLS cases may reflect conceptual design rather than diagnostic failure. In our cohort, AC possible and rEEC possible correctly diagnosed more patients either with bulbar-onset ALS or the PBP subtype than GCC, while previous studies reported no significant differences and a comparably high sensitivity (Hannaford et al., 2021, Pugdahl et al., 2021). PBP is often described as an UMN dominant phenotype (Pinto et al., 2019). Since exclusive UMN signs are not represented in the GCC, a higher sensitivity of the rEEC and AC possible seems reasonable. When excluding the PBP subtype from the sensitivity analysis of bulbar onset ALS in our cohort, no significant difference of sensitivity remains across the diagnostic criteria sets (data not shown). However, the clinical definition of PBP remains inconsistent. Our findings still demonstrated a consistent high sensitivity of the GCC in the context of bulbar ALS.

The addition of neurofilaments as biomarkers in the previous diagnostic criteria did not improve sensitivity compared to the GCC. This highlights the clinical relevance of the GCC even in the era of biomarker profiling. Considering the false-negative cases according to GCC, the analysis of neurofilament levels in patients with bulbar onset and pure UMN involvement may be helpful for establishing ALS diagnosis: Two of three false-negative cases presented with elevated neurofilament levels. Because of the small sample size, concluding statements cannot be made at the moment. Future studies may show, if and how neurofilaments could be incorporated as additional diagnostic criteria. The neurofilament thresholds applied in this study (>45 pg/ml serum NfL; >560 pg/ml CSF pNfH) were adopted from previously published reference cohorts (Steinacker et al., 2016; Verde et al., 2019) without internal validation, as establishing new reference values was beyond the scope of this study. Since absolute neurofilament values depend on the analytical platform and laboratory protocol used (Behzadi et al., 2021), these cut-offs are not universally transferable and should be regarded as orientation values. Confirmation in independent prospective cohorts would support the broader clinical use of these thresholds.

The interpretation of specificity in our cohort is not reliable due to the small number of patients who were not diagnosed with ALS. A Chinese and an Italian study, also conducted at specialized ALS centers, examined similar cohorts with equally few non-ALS patients (Ferullo et al., 2024; Shen et al., 2021). Consequently, the post-test probability is high at more than 99% for all diagnostic criteria sets in our setting. While this considerably limits interpretation of sensitivity values, it nevertheless reflects an accurate representation of the clinical reality in a specialized ALS clinic and underlines the character of our study as sensitivity-focused analysis in a high-pretest-probability cohort. Additionally, it highlights the need for more robust estimates of specificity through validation in larger, independent datasets. It can generally be observed that the specificity of the GCC appears to be lower than that of the rEEC and AC (von Quednow et al., 2025). This difference aligns with the simplified nature of the GCC, which ultimately results in a higher number of individuals meeting the diagnostic threshold. In our cohort, there were three false positives according to the GCC. All three were male and, unsurprisingly, had an LMN-dominant phenotype in common. This emphasizes the importance of differential diagnostics, which remain an integral part of the diagnostic process and clinical follow-up. While the GCC clearly help to improve early diagnosis of ALS, they can be non-specific in certain clinical scenarios (with overlapping symptoms or in particularly slowly progressive or focal presentations). The primary limitation of the rEEC and AC, on the other hand, lies in their complexity, which involves multiple diagnostic categories and is prone to high interobserver variability (Johnsen et al., 2019).

Beyond improved sensitivity, the GCC have practical advantages for the diagnostic workflow. The dichotomous structure simplifies EMG interpretation and documentation: equivocal findings no longer need to be classified as possible, probable, or definite. Because UMN and LMN involvement in a single region is sufficient, an extensive multi-region EMG protocol is also no longer required for diagnosis. The binary classification is also easier to communicate to patients – although the earlier criteria were not intended for this purpose, the gradated labels often suggested uncertainty despite high clinical suspicion and triggered repeated evaluations before disease progression forced reclassification (Traynor et al., 2000).

The analysis of NfL and pNfH levels confirmed previous findings that these biomarkers could play a potentially valuable role in the differentiation of ALS subtypes. The highest NfL levels were measured in PBP and in subtypes with dominant LMN involvement, such as PMA and FAS. These findings are consistent with evidence suggesting that neurofilaments are released from the root segments of the spinal cord, which may explain the elevated NfL levels observed in subtypes with prominent lower motor neuron involvement, due to the affection of the peripheral nervous system (Meyer et al., 2023). In contrast, the PLS subtype showed significantly lower NfL levels, reflecting pure UMN affection in this subtype. pNfH levels were similarly distributed in our study, with PBP and FAS subtypes showing the highest values. Patients with PLS also had the lowest values here, indicating a lower sensitivity of pNfH for early stages of PLS (Zucchi et al., 2020). As the degree of neurofilament elevation is linked to prognosis and survival, lower neurofilament levels reflect the favorable prognosis of PLS regarding progression and survival of ALS, as previously reported (Meyer et al., 2024).

Highest NfL levels were observed in bulbar onset ALS, followed by cervical and lumbar onset patients, which is in line with previous studies (Meyer et al., 2024). Bulbar onset, which typically presents with more widespread or earlier involvement of UMN and LMN, is associated with higher neurofilament levels compared to more localized forms, such as lumbar onset (Behzadi et al., 2021; Zecca et al., 2022). This variation in neurofilament levels depending on clinical onset could potentially help differentiate between different disease patterns within in the OPM classification.

The limited number of patients without an ALS diagnosis in our study arises from the inclusion criteria, which focused on suspected ALS cases. GCC are typically applied only to suspected ALS patients in a clinical setting. Consequently, while the small number of controls limits specificity assessments, limits generalizability and poses a risk of selection bias, the study population still reflects clinical reality. Patients are typically pre-selected when they are referred to a specialized ALS center, having often been initially assessed by outpatient neurologists before being referred to a tertiary clinic for assessment and/or to obtain a second opinion. Since the same clinical and electrophysiological information was considered when applying the rEEC, AC and GCC and when determining the final diagnosis, incorporation bias is inherent in our study. This may have led to higher sensitivity estimates of all the diagnostic criteria. Because the bias affects them equally, the relative comparison between rEEC, AC and GCC remains valid. Further limitations of our study are the retrospective and monocentric design, limiting generalizability of our findings, and the incomplete follow up data. Follow-up was not available in many cases, as patients were referred to our clinic from a large catchment area in north-west Germany and sought further care with local healthcare providers. However, the monocentric design allowed for comparability through standardized protocols in a large cohort.

## 5 Conclusion

In summary, the sensitivity of the GCC in our high-pretest-probability cohort is higher than that of the rEEC, AC, the rEEC possible and AC possible, and the rEEC-plus and AC-plus incorporating neurofilaments into the diagnostic process. They are well suited for the application in a specialized ALS clinic and may facilitate early diagnosis of ALS. ALS is a spectrum ranging from LMN to UMN involvement. The GCC cover PMA but not PLS. Biomarkers such as neurofilaments could be used to refine the diagnostic criteria, ensuring coverage of the entire ALS spectrum. Prospective multicenter studies are needed to confirm these findings and to further define the role of biomarkers within diagnostic criteria for ALS.

## Contribution

C.K., C.W. and L.H.V. wrote the manuscript, L.H.V. und S.K. planned and designed the study, C.K. and C.W. performed data collection and interpretation, C.K. and L.H.V. performed statistics, S.K. and L.H.V. coordinated the study. A.H., S.K. and S.P. were responsible for local data collection and critically revised the manuscript. A.H., S.P. and S.K. were involved in coordination of the study and critically revised the manuscript.

## Declaration of competing interest

The authors declare the following financial interests/personal relationships which may be considered as potential competing interests: C.K., A.H., S.K. and L.H.V. declare that they have no known competing financial interests or personal relationships that could have appeared to influence the work reported in this paper. C.W. received compensation for travel expenses from ITF Pharma GmbH outside of the submitted work. S.P. has received speaker fees, non-financial support and research support from Biogen, Roche, Amylyx, Cytokinetics, Ferrer, ITF Pharma, Zambon and Sanofi and served on advisory boards of Amylyx, Biogen, Roche, Zambon and ITF Pharma outside of the submitted work.

## Glossary

AC: Awaji criteria. AC poss: Awaji criteria including the possible category. AC-plus: Awaji criteria with incorporated neurofilament values. ALS: amyotrophic lateral sclerosis. ALSFRS-R: ALS Functional Rating Scale-Revised. CI: confidence intervals. CSF: cerebrospinal fluid. ELISA: enzyme-linked immunosorbent assay. EMG: electromyography. FAS: Flail-arm syndrome. FP: false positive. FN: False negative. GCC: Gold Coast criteria. LMN: lower motor neuron. NfL: neurofilament light chain. PBP: progressive bulbar paralysis. PMA: Progressive Muscular Atrophy. PLS: Primary Lateral Sclerosis. pNfH: phosphorylated neurofilament heavy chain. rEEC: revised El Escorial criteria. rEEC poss: revised El Escorial criteria including the possible category. rEEC-plus: revised El Escorial criteria with incorporated neurofilament values. Se: Sensitivity. Sp: Specificity. TN: True negative. TP: true positive. UMN: upper motor neuron.

## Appendix A Supplementary data

Supplementary material Diagnostic accuracy of the diagnostic criteria sets in the subgroups (disease duration < or > 18 months, site of onset, subtype).
