# Supplementary material for: Gold coast criteria but not neurofilaments improve diagnostic sensitivity in a specialized ALS university center
Source: Clin Neurophysiol Pract. 2026 Jun 12;11:512–21. doi: 10.1016/j.cnp.2026.06.004 (PMC13459124; doi:10.1016/j.cnp.2026.06.004)
Supplement: Supplementary file 1 [file mmc1.docx]

**Supplementary Material**

| **Subgroup analysis** | | | | | | | |
| --- | --- | --- | --- | --- | --- | --- | --- |
|  | **disease duration < 18 months** | | | | | | |
|  | **TP** | **FP** | **TN** | **FN** | **Se (%)** | **95%CI** | **p-value** |
| GCC | 279 | 3 | 1 | 21 | 93.0 | 89.5-95.4 |  |
| AC | 220 | 2 | 2 | 80 | 73.3 | 68.1-78.0 | <0.001 |
| AC poss | 256 | 2 | 2 | 44 | 85.3 | 80.9-88.9 | 0.001 |
| rEEC | 214 | 2 | 2 | 86 | 71.3 | 66.0-76.2 | <0.001 |
| rEEC poss | 255 | 2 | 2 | 45 | 85.0 | 80.5-88.6 | <0.001 |
|  | **disease duration > 18 months** | | | | | | |
| GCC | 115 | 0 | 1 | 11 | 91.3 | 85.0-95.1 |  |
| AC | 89 | 0 | 1 | 37 | 70.6 | 62.2-77.9 | <0.001 |
| AC poss | 103 | 1 | 0 | 23 | 81.7 | 74.1-87.5 | 0.052 |
| rEEC | 89 | 0 | 1 | 37 | 70.6 | 62.2-77.9 | <0.001 |
| rEEC poss | 103 | 1 | 0 | 23 | 81.7 | 74.1-87.5 | 0.052 |
|  | **onset: bulbar** | | | | | | |
| GCC | 125 | 1 | 0 | 17 | 88.0 | 81.7-92.4 |  |
| AC | 114 | 1 | 0 | 28 | 80.3 | 73.0-86.0 | 0.003 |
| AC poss | 134 | 1 | 0 | 8 | 94.4 | 89.3-97.1 | 0.049 |
| rEEC | 105 | 1 | 0 | 37 | 73.9 | 66.2-80.5 | <0.001 |
| rEEC poss | 133 | 1 | 0 | 9 | 93.7 | 88.4-96.6 | 0.096 |
|  | **onset: cervical** | | | | | | |
| GCC | 122 | 1 | 0 | 5 | 96.1 | 91.1-98.3 |  |
| AC | 83 | 0 | 1 | 44 | 65.4 | 56.7-73.1 | <0.001 |
| AC poss | 94 | 0 | 1 | 33 | 74.0 | 65.8-80.9 | <0.001 |
| rEEC | 84 | 0 | 1 | 43 | 66.1 | 57.5-73.8 | <0.001 |
| rEEC poss | 94 | 0 | 1 | 33 | 74.0 | 65.8-80.9 | <0.001 |
|  | **onset: lumbal** | | | | | | |
| GCC | 134 | 1 | 2 | 10 | 93.1 | 87.7-96.2 |  |
| AC | 100 | 1 | 2 | 44 | 69.4 | 61.5-76.4 | <0.001 |
| AC poss | 119 | 2 | 1 | 25 | 82.6 | 75.6-88.0 | 0.009 |
| rEEC | 102 | 1 | 2 | 42 | 70.8 | 62.9-77.6 | <0.001 |
| rEEC poss | 119 | 2 | 1 | 25 | 82.6 | 75.6-88.0 | 0.009 |
|  | **onset: thoracic** | | | | | | |
| GCC | 13 | 0 | 0 | 0 | 100.0 | 77.2-100 |  |
| AC | 12 | 0 | 0 | 1 | 92.3 | 66.7-98.6 | n.c. |
| AC poss | 13 | 0 | 0 | 0 | 100% | 77.2-100 | n.c. |
| rEEC | 12 | 0 | 0 | 1 | 92.3 | 66.7-98.6 | n.c. |
| rEEC poss | 12 | 0 | 0 | 1 | 92.3 | 66.7-98.6 | n.c. |
|  | **subtype: PBP** | | | | | | |
| GCC | 27 | 0 | 0 | 8 | 77.1 | 61.0-87.9 |  |
| AC | 25 | 0 | 0 | 10 | 71.4 | 54.9-83.7 | 0.5 |
| AC poss | 33 | 0 | 0 | 2 | 94.3 | 81.4-98.4 | 0.031 |
| rEEC | 19 | 0 | 0 | 16 | 54.3 | 38.2-69.5 | 0.008 |
| rEEC poss | 33 | 0 | 0 | 2 | 94.3 | 81.4-98.4 | 0.031 |
|  | **subtype: Flail-arm syndrome** | | | | | | |
| GCC | 26 | 0 | 0 | 1 | 96.3 | 81.7-99.3 |  |
| AC | 17 | 0 | 0 | 10 | 63.0 | 44.2-78.5 | 0.004 |
| AC poss | 19 | 0 | 0 | 8 | 70.4 | 51.5-84.1 | 0.039 |
| rEEC | 17 | 0 | 0 | 10 | 63.0 | 44.2-78.5 | 0.004 |
| rEEC poss | 19 | 0 | 0 | 8 | 70.4 | 51.5-84.1 | 0.039 |
|  | **subtype: PMA** | | | | | | |
| GCC | 25 | 0 | 0 | 1 | 96.2 | 81.1-99.3 |  |
| AC | 4 | 0 | 0 | 22 | 15.4 | 6.1-33.5 | <0.001 |
| AC poss | 5 | 0 | 0 | 21 | 19.2 | 8.5-37.9 | <0.001 |
| rEEC | 5 | 0 | 0 | 21 | 19.2 | 8.5-37.9 | <0.001 |
| rEEC poss | 5 | 0 | 0 | 21 | 19.2 | 8.5-37.9 | <0.001 |
|  | **subtype: PLS** | | | | | | |
| GCC | 6 | 0 | 1 | 11 | 35.3 | 17.3-58.7 |  |
| AC | 5 | 0 | 1 | 12 | 29.4 | 13.3-53.1 | 1.0 |
| AC poss | 14 | 1 | 0 | 3 | 82.4 | 59.0-93.8 | 0.004 |
| rEEC | 7 | 0 | 1 | 10 | 41.2 | 21.6-64.0 | 1.0 |
| rEEC poss | 13 | 1 | 0 | 4 | 76.5 | 52.7-90.4 | 0.021 |
|  | **subtype: classic** | | | | | | |
| GCC | 290 | 1 | 0 | 8 | 97.3 | 94.8-98.6 |  |
| AC | 248 | 1 | 0 | 50 | 83.2 | 78.6-87.0 | <0.001 |
| AC poss | 276 | 1 | 0 | 22 | 92.6 | 89.1-95.1 | 0.007 |
| rEEC | 245 | 1 | 0 | 53 | 82.2 | 77.5-86.1 | <0.001 |
| rEEC poss | 276 | 1 | 0 | 22 | 92.6 | 89.1-95.1 | 0.007 |
|  | **subtype: other** | | | | | | |
| GCC | 20 | 2 | 1 | 3 | 87.0 | 67.9-95.5 |  |
| AC | 10 | 1 | 2 | 13 | 43.5 | 25.6-63.2 | <0.001 |
| AC poss | 12 | 1 | 2 | 11 | 52.2 | 33.0-70.8 | 0.012 |
| rEEC | 10 | 1 | 2 | 13 | 43.5 | 25.6-63.2 | <0.001 |
| rEEC poss | 12 | 1 | 2 | 11 | 52.2 | 33.0-70.8 | 0.012 |

**Table S1**. Diagnostic accuracy of the diagnostic criteria sets in the subgroups (disease duration < or > 18 months, site of onset, subtype). McNemar’s test was applied to test for differences in sensitivity compared to the GCC and are demonstrated with 95% confidence intervals. P-values < 0.05 were considered significant. Calculation of Specificity was not calculated when the sample size was 0 or 1. Test for statistic difference in specificity was not performed due to the small number of true negative and false positive cases. AC: Awaji criteria. AC poss: Awaji criteria including the possible category. AC-plus: Awaji criteria with incorporated neurofilament values. CI: confidence intervals. FAS: Flail-arm syndrome. FP: false positive. FN: False negative. GCC: Gold Coast criteria. PBP: progressive bulbar paralysis. PMA: Progressive Muscular Atrophy. PLS: Primary Lateral Sclerosis. rEEC: revised El Escorial criteria. rEEC poss: revised El Escorial criteria including the possible category. rEEC-plus: revised El Escorial criteria with incorporated neurofilament values. Se: Sensitivity. TN: True negative. TP: true positive.
